# Role of MIRU-VNTR and spoligotyping in assessing the genetic diversity of *Mycobacterium tuberculosis* in Henan Province, China

**DOI:** 10.1186/s12879-018-3351-y

**Published:** 2018-09-03

**Authors:** Jie Shi, Danwei Zheng, Yankun Zhu, Xiaoguang Ma, Shaohua Wang, Hui Li, Jin Xing

**Affiliations:** Henan Province Center for Disease Control and Prevention, Zheng Zhou, 450016 Henan People’s Republic of China

**Keywords:** *Mycobacterium tuberculosis*, MIRU-VNTR, Spoligotyping, Henan Province

## Abstract

**Background:**

Tuberculosis remains a serious threat to human health as an infectious disease in China. Henan, a most populated province in China, has a high incidence of tuberculosis (TB). Though the genetic diversity of *Mycobacterium tuberculosis* (MTB) has been investigated in many regions, there have been only a few studies on the molecular characteristics and drug resistance phenotypes in Henan. This is the first study on the genetic profile of MTB from Henan.

**Methods:**

A total of 668 MTB isolates from various areas were genotyped with spoligotyping and 26-locus MIRU-VNTR (classical 24-locus MIRU-VNTR and 2 other loci). The association between TB spoligotype signatures and drug-resistant profiles was analysed.

**Results:**

Our data revealed that MTB isolates circulating in Henan had a high degree of genetic variation. The Beijing family was the most predominant genotype (83.53%,*n* = 558), and the typical Beijing type(ST1) was the major sublineage (81.73%,*n* = 546). In total,668 isolates were divided into 567 different types, forming 38 clusters (2–15 isolates per cluster), and 529 unique types by 26-locus MIRU-VNTR analysis. There was no correlation between the Beijing family and gender, age at diagnosis or treatment history, whereas the Beijing family was significantly associated with all four first-line drug resistance and multidrug-resistant phenotypes. For these samples, 15 of 26 MIRU-VNTR loci had high or moderate discriminatory power according to the Hunter-Gaston discriminatory index. A combination of the 10 most polymorphic loci had similar discriminatory power as the 26-locus set.

**Conclusion:**

The Beijing genotype is the most prevalent family. Ten-locus MIRU-VNTR in combination with spoligotyping can efficiently classify the molecular type of MTB in Henan Province.

**Electronic supplementary material:**

The online version of this article (10.1186/s12879-018-3351-y) contains supplementary material, which is available to authorized users.

## Background

Tuberculosis (TB), caused by *Mycobacterium tuberculosis*(MTB), remains a significant public health problem worldwide. It is estimated that approximately one-third of the world’s population has been infected with MTB, and 1.8 million people die of this disease annually. Among the 22 high TB burden countries reported by the World Health Organization, China ranks second in the world with approximately 1.3 million new cases [1, 2].

Recently, molecular epidemiology tools have been used to assess risk factors associated with recent transmissions [3],to track infection transmission dynamics, to distinguish relapse or reinfection and to detect suspected outbreaks; therefore, these tools play a critical role in tuberculosis research and control. MTB molecular markers promoted the development of reproducible genotyping methods [4], including insertion sequence 6110 (IS6110), restriction fragment length polymorphism (RFLP) typing [5], spacer oligonucleotide typing (spoligotyping) [6], single nucleotide polymorphism analysis [7],mycobacterial interspersed repetitive unit variable number tandem repeats (MIRU-VNTRs) assessment [8], large sequence polymorphism (LSP) typing [9], and genome [10] sequence analysis. IS6110-RFLP has been the gold standard for genotyping MTB since 1993, but this procedure is time consuming, technically demanding and labour intensive. This method also requires about one microgram of high-quality DNA. Moreover, the discriminating efficiency of this method is insufficient for strains harbouring low copy numbers of IS6110.The Beijing genotype strains exhibit highly similar RFLP patterns, and therefore, discrimination among them is difficult. In addition, rapid and inexpensive genotyping methods based on PCR, such as MIRU-VNTR and spoligotyping, have been effectively used to investigate the genetic relationships and epidemiological characteristics of Beijing strains.

Non-coding regions of the MTB genome contain a set of identical 36-bp direct repeats (DRs), which are separated by 35- to 41-bp unique DNA spacer sequences. Spoligotyping, a rapid and highly reproducible method, detects the presence or absence of DR loci [11]. The results can be represented in a simple binary format that enables the construction of large-scale databases [12].Therefore, it is considered the gold standard for identifying the Beijing family strains, which have lack spacers 1 to 33 and harbour spacers 34–43 in the DR region [11, 13]. Unfortunately, spoligotyping remains less discriminatory, especially in regions with a high prevalence of Beijing isolates [14].The discriminatory power is improved when spoligotyping is combined with VNTR.

MIRU-VNTR, a new PCR-based typing method, determines the size and repeated number of units in each locus by amplifying mycobacterial interspersed repetitive units. Easy operation, economical cost, reproducible results and high discriminatory power make it practical for routine use [15], and the digital results from this method can be compared and exchanged easily between different laboratories [16, 17]. Twelve-locus MIRU-VNTR has been widely used in most cases but has lower discrimination for the Beijing family [18]. Nevertheless, the 24- and 15-locus sets effectively improved discrimination compared with the initial 12-locus set [19].

In China, more than 80% of tuberculosis patients are in rural communities [20]. Henan Province, the most highly populated province in China, has a significantly higher proportion of the population living in rural areas. Therefore, the epidemic situation of tuberculosis in Henan remains severe. The numbers of both TB and drug-resistant TB patients in Henan are larger than those in any other province, and tuberculosis and HIV co-infection make the bad situation worse. Thus, study of a *M.tuberculosis* transmission model can help determine risk factors and improve contact tracing. Moreover, little was known about the genetic diversity of MTB in this region until now. The study is the first to use26-locus MIRU-VNTR, including the standard 24-locus and two other loci (ETRF and Mtub38) [21, 22]},for assessments in Henan. In this study, we carried out spoligotyping and MIRU-VNTR to classify 668 representative strains from17 cities. The objective of this study was to assess the diversity of MTB circulating in Henan with higher discrimination and to analyse the probable association between drug resistance profiles and genotypes.

## Methods

### *M. tuberculosis* clinical isolate collection

In total, 668 strains of MTB were collected from smear-positive pulmonary TB patients in the Tuberculosis Control Institution from various regions of Henan Province during 2015. The patients were from the following regions: 70 from Zhengzhou, 70 from NanYang, 65 from Zhoukou, 50 from Zhumadian, 50 from Luoyang,50 from Kaifeng, 50 from Shangqiu, 45 from Xinyang, 40 from Xinxiang, 35 from Pingdingshan, 35 from Anyang, 20 from Puyang, 19 from Luohe, 18 from Xuchang,18 from Jiaozuo,18 from Sanmenxia, 15 from Hebi, and none from Jiyuan. *M. tuberculosis* H37Rv was used as the control strain.

### Ethics statement

This project was approved by the Ethics Review Committee of Henan CDC. All the patients with pulmonary TB provided informed consent before participation in this investigation. Ethics were respected throughout the whole study period.

### Genomic DNA extraction

All the isolates were cultured in Lowenstein-Jensen (L-J) medium for 3–4 weeks at 37 °C. A loopful of colonies was added to 300 μl of TE buffer at pH 8.0. The suspension of bacterial cells was incubated at 85 °C for 30 min to inactivate pathogens, followed by centrifugation at 13,000 *g* for 5 min. The supernatant containing DNA was used as a template for PCR.

### Spoligotyping

These collected isolates were subjected to spoligotyping on commercially available membranes according to a previously described standard protocol [23–25]. Briefly, the direct repeat (DR) regions were amplified with biotin-labelled Dra and Drb primers [11], and then, the amplicons were hybridized with a nylon membrane that covalently bound a set of 43 oligonucleotide probes [11]. The hybrid membranes were washed with SSPE buffer containing 0.5%SDS and incubated with streptavidin peroxidase conjugate. The results were visualized with the ECL system. The spoligotyping results in octal format were compared with those in the international spoligotyping database SITVIT2 to assign the Spoligotype (or Shared) International Type(SIT) codes [26].

### MIRU-VNTR typing

MIRU-VNTR typing based on 26 loci, including the standard 24 loci [27] and 2 other loci, i.e., ETRF and Mtub38 [21], was performed to determine genetic relationships among isolates in our study. First, each locus was amplified individually as previously described [22]. Then, the PCR products were detected in a 1.5% agarose gel using a 50 bp DNA ladder as the molecular weight standard. The number of tandem repeats was calculated based on the length of the repeat and flank sequences for each locus. The PCR products of H37Rv were loaded to ensure accuracy and the PCR products of sterile water were used to control for reagent contamination. To visualize evolutionary relationships among these clinical isolates, the resulting data were analysed by BioNumerics 6.6 as a characteristic data set. The results of MIRU-VNTR were analysed to construct a dendrogram based on the UPGMA algorithm. The discriminatory power of each locus was evaluated using the Hunter and Gaston Discriminatory Index (HGDI) [28].The Hunter-Gaston Index (HGDI) was calculated with the equation:

$$ HGDI\kern0.5em =\kern0.5em 1-\frac{1}{N\kern0.5em \left(N-1\right)}\sum \limits_{j=1}^s{n}_j\left({n}_j-1\right) $$

### Strain isolation and drug susceptibility test

Epidemiological information, such as age, sex, and clinical treatment history of the patients, was collected with a questionnaire method.

The sputum samples of clinical patients were isolatedand cultured using Lowenstein-Jensen (L-J) media. Then, the drug susceptibility tofirst- and second-line anti-TB drugs was determined using the proportion method with H37Rv as a control [13]. The drug concentrations in L-J media were as follows: isoniazid (INH) 0.2 μg/ml, rifampicin (RIF) 40 μg/ml, ethambutol (EMB) 2 μg/ml, streptomycin (SM) 4 μg/ml, kanamycin (KM) 3 μg/ml, ofloxacin (OFX) 2 μg/ml, capreomycin (CPM) 40 μg/ml, p-aminosalicylic acid(PAS) 1 μg/ml,and prothionamide (Pto) 40 μg/ml. MDR-TB (multidrug resistance-TB) is defined as an isolate that isresistant to both isoniazid and rifampicin. Extensivedrug-resistant tuberculosis (XDR-TB) is defined as an MDR isolate that isalso resistant to any fluoroquinolone and at least one of three injectable second-line drugs, such as KM or CPM. In addition, MDR isolates resistant to only OFX or KM are defined as pre-XDR-TB [14]}.

### Data analysis

The Hunter-Gaston discriminatory Index (HGDI) was calculated as previously described to assess allelic diversity at VNTR loci [29]. The data were analysed with SPSS 19.0 software. The statistical associations between genotype and drug susceptibility, age, gender, and treatment history were detected using a Chi-squared test or Fisher’s exact test. A 5% level of significance (*p* ≤ 0.05) was considered statistically significant.

## Results

### Spoligotyping

In this study, 668 isolates were clustered into 35 distinct genotypes by spoligotyping. The octal coded spoligotyping results were assigned spoligotyping shared international type (SIT) numbers and then compared with those in spolDB4 [26] and theupdated SITVIT [30] databases. In total, 643 isolates grouped into 10 clusters comprising 2 to 546 isolates, while the other 25 isolates showed an orphan spoligotyping pattern. Among all 668 isolates, 8(1.19%) could not be matched to any determined patterns in either database and thus were labelled “unknown”. The Beijing family genotypes strongly predominated in our area, accounting for 558/668 (83.53%) of the isolates, followed by the T1 family with 59/668isolates (8.83%) and the MANU2 family with 20/168isolates (2.99%). The genotypes Beijing, T1 and MANU2 were dominant, accounting for 95.35% of the total. Among the 558 Beijing isolates, 546 showed the typical Beijing family pattern: the absence of the first 34 spacers and the presence of spacers 35 to 43 [28]. The remaining 12 isolates lacked one or more spacers that are present in the typical Beijing pattern and thus were classified as Beijing-like or an atypical Beijing family. The other 110 non-Beijing isolates were subdivided into 28 lineages. The high clustering rate (94.7%) of spoligotyping was in concordance with the lower discrimination for the Beijing family (Table 1).

**Table 1:** Spoligotypes shared by *M. tuberculosis* strains evaluated in this study

Octonary code of Spoligotype	Spoligotyping	Family^a^	SIT^b^	No.^c^	Prevalence^d^
000000000003771	□□□□□□□□□□□□□□□□□□□□□□□□□□□□□□□□□□■■■■■■■■■	BEIJING	1	546	81.74%
000000000003571	□□□□□□□□□□□□□□□□□□□□□□□□□□□□□□□□□□■■■□■■■■■	BEIJING-LIKE	632	2	0.30%
000000000003731	□□□□□□□□□□□□□□□□□□□□□□□□□□□□□□□□□□■■■■■□■■■	BEIJING-LIKE	190	2	0.30%
000000000000071	□□□□□□□□□□□□□□□□□□□□□□□□□□□□□□□□□□□□□□□■■■■	BEIJING-LIKE	ORPHAN	1	0.15%
000000000003661	□□□□□□□□□□□□□□□□□□□□□□□□□□□□□□□□□□■■■■□■■□■	BEIJING-LIKE	1651	1	0.15%
000000000003751	□□□□□□□□□□□□□□□□□□□□□□□□□□□□□□□□□□■■■■■■□■■	BEIJING-LIKE	941	1	0.15%
000000000000771	□□□□□□□□□□□□□□□□□□□□□□□□□□□□□□□□□□□□■■■■■■■	BEIJING-LIKE	269	5	0.75%
777,777,760,020,771	■■■■■■■■■■■■■■■■■■■■■■■□□□□□□□□■□□□□■■■■■■■	H3	791	1	0.15%
777,777,777,020,771	■■■■■■■■■■■■■■■■■■■■■■■■■■■□□□□■□□□□■■■■■■■	H3		1	0.15%
000000007760771	□□□□□□□□□□□□□□□□□□□□□□□□■■■■■■■■□□□□■■■■■■■	LAM3 and S /convergent	4	1	0.15%
777,777,777,613,771	■■■■■■■■■■■■■■■■■■■■■■■■■■■■■□□□■□■■■■■■■■■	MANU1	ORPHAN	1	0.15%
777,777,777,763,771	■■■■■■■■■■■■■■■■■■■■■■■■■■■■■■■■□□■■■■■■■■■	MANU2	54	16	2.40%
757,777,777,763,771	■■■■□■■■■■■■■■■■■■■■■■■■■■■■■■■■□□■■■■■■■■■	MANU2	ORPHAN	2	0.30%
777,737,777,763,771	■■■■■■■■■■■■□■■■■■■■■■■■■■■■■■■■□□■■■■■■■■■	MANU2	583	1	0.15%
777,777,577,763,771	■■■■■■■■■■■■■■■■■■■□■■■■■■■■■■■■□□■■■■■■■■■	MANU2	ORPHAN	1	0.15%
576,377,777,760,771	■□■■■■■■□□■■■■■■■■■■■■■■■■■■■■■■□□□□■■■■■■■	S	1211	1	0.15%
777,777,777,760,771	■■■■■■■■■■■■■■■■■■■■■■■■■■■■■■■■□□□□■■■■■■■	T1	53	54	8.08%
037777777760771	□□□□■■■■■■■■■■■■■■■■■■■■■■■■■■■■□□□□■■■■■■■	T1	272	1	0.15%
577,777,777,760,771	■□■■■■■■■■■■■■■■■■■■■■■■■■■■■■■■□□□□■■■■■■■	T1	334	1	0.15%
777,703,777,760,771	■■■■■■■■■■■■□□□□■■■■■■■■■■■■■■■■□□□□■■■■■■■	T1	102	1	0.15%
777,777,437,760,771	■■■■■■■■■■■■■■■■■■■□□□■■■■■■■■■■□□□□■■■■■■■	T1	1051	1	0.15%
777,777,777,760,631	■■■■■■■■■■■■■■■■■■■■■■■■■■■■■■■■□□□□■■□□■■■	T1	888	1	0.15%
777,777,777,760,731	■■■■■■■■■■■■■■■■■■■■■■■■■■■■■■■■□□□□■■■□■■■	T2	52	6	0.90%
577,777,777,760,731	■□■■■■■■■■■■■■■■■■■■■■■■■■■■■■■■□□□□■■■□■■■	T2	1302	1	0.15%
777,737,777,760,771	■■■■■■■■■■■■□■■■■■■■■■■■■■■■■■■■□□□□■■■■■■■	T3	37	8	1.20%
577,737,777,760,771	■□■■■■■■■■■■□■■■■■■■■■■■■■■■■■■■□□□□■■■■■■■	T3	ORPHAN	2	0.30%
777,777,777,760,011	■■■■■■■■■■■■■■■■■■■■■■■■■■■■■■■■□□□□□□□□□■■	U		1	0.15%
563,777,777,760,771	■□■■■□□■■■■■■■■■■■■■■■■■■■■■■■■■□□□□■■■■■■■	unknown^e^		1	0.15%
577,777,403,760,771	■□■■■■■■■■■■■■■■■■■□□□□□□■■■■■■■□□□□■■■■■■■	unknown		1	0.15%
703,777,760,000,771	■■■□□□□■■■■■■■■■■■■■■■■□□□□□□□□□□□□□■■■■■■■	unknown		1	0.15%
703,777,760,063,771	■■■□□□□■■■■■■■■■■■■■■■■□□□□□□□■■□□■■■■■■■■■	unknown		1	0.15%
741,777,777,760,731	■■■■□□□□■■■■■■■■■■■■■■■■■■■■■■■■□□□□■■■□■■■	unknown		1	0.15%
777,660,007,460,771	■■■■■■■■■■■□■■□□□□□□□□□□■■■■□□■■□□□□■■■■■■■	unknown		1	0.15%
777,777,307,760,731	■■■■■■■■■■■■■■■■■■□■■□□□■■■■■■■■□□□□■■■□■■■	unknown		1	0.15%
777,777,775,760,631	■■■■■■■■■■■■■■■■■■■■■■■■■□■■■■■■□□□□■■□□■■■	unknown		1	0.15%

^a^Representing spoligotype families annotated in SITVITWEB database

^b^SIT from SITVITWEB database

^c^Number of strains with the same SIT

^d^Prevalence represents the percentage of isolates with a common SIT among all isolates in this study

^e^unknown represents the spoligotyping type which is not found in SITVITWEB database

**Fig. 1:**
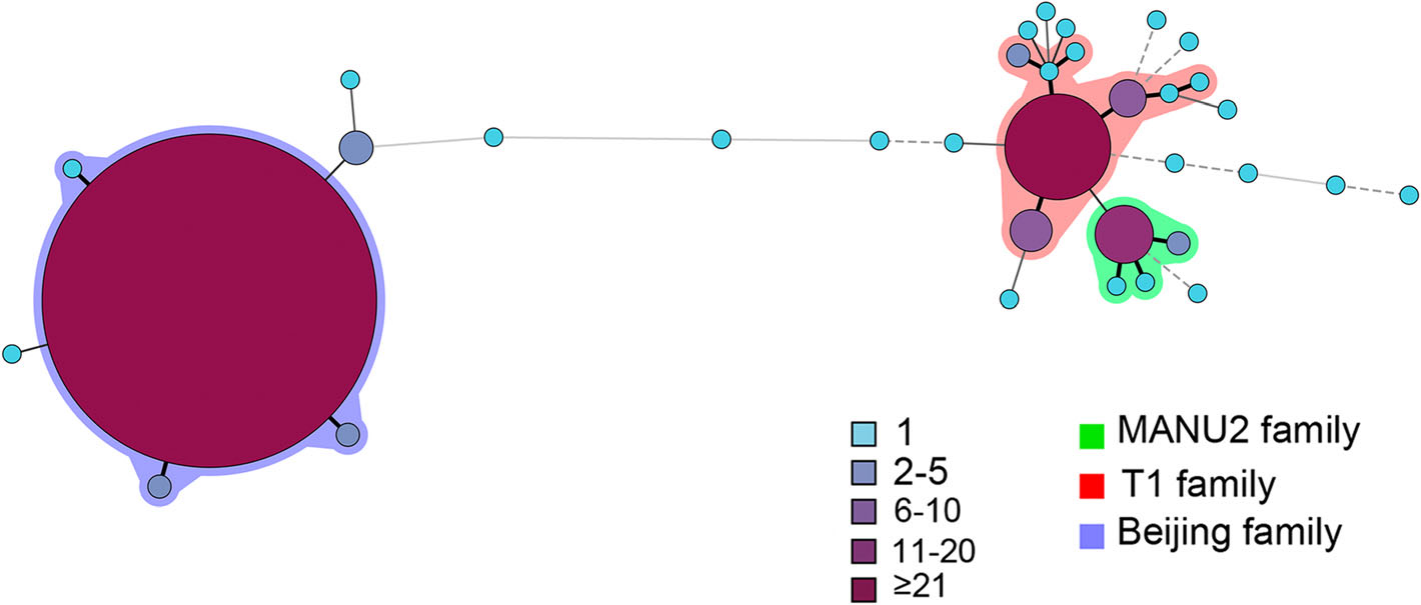
Minimum spanning tree showing clustering by spoligotyping of 668 *M.tuberculosis* isolates from Henan. Each nodal point represents a cluster with identical genotypes, and the colour and size of the nodal points are relative to the number of strains within that cluster. Different colours are assigned to the different groups, and the colour code is indicated on the side

To evaluate the genetic relations among all 668 isolates, the minimum spanning tree was generated using BioNumerics 6.6 software. Each node represents a distinct genotype, and the size of each node depends on the number of the corresponding cluster [31]. These isolates were divided into two major lineages, the Beijing family and the non-Beijing family. The largest cluster contained 546 typical Beijing family isolates (SIT1) with small clusters of the atypical Beijing family around it. The two larger clusters on the right comprised the T1 and MANU2 families and were surrounded by other non-Beijing families and 8 unknown genotypes. Therefore, the results suggested that the unknown genotyped isolates maybe genetically closer to the non-Beijing family (Fig. 1).

### 26-locus MIRU-VNTR

The analysis of 26-locus MIRU-VNTR showed that 139 isolates (23.9%) grouped into 38 clusters, and the remaining 529 isolates shared unique patterns. The largest cluster contained 15 strains, and the other clusters consisted of 2–14 isolates. The clustering rate of 26-locus MIRU-VNTR was 15.1%. There was some discrepancy between the spoligotyping and 26-locus MIRU-VNTR for the Beijing family isolates. The largest cluster of the MIRU-VNTR mainly included Beijing family isolates; however, 33 isolates belonged to the non-Beijing family patterns in this largest clade (Additional file 1: Figure S1). This finding might be due to mixed infection of two different TB isolates in these patients.

To evaluate the allelic diversity of 26-locus MIRU-VNTR among the isolates in this study, we calculated the Hunter-Gaston discriminatory index (HGDI) for each locus. As previously reported, MIRU-VNTR loci were further designated highly (> 0.6), moderately (0.3–0.6), or poorly (< 0.3) discriminatory loci according to the HGDI scores [32]. These loci in our study exhibited significantly different discriminatory powers with various HGDI scores from 0.767 for Qub11b to 0.015 for MIRU24 (Table 2). Among all the strains, four loci were designated highly discriminatory loci. Qub11b had the best discriminatory power(HGDI = 0.767), followed by Mtub21 (HGDI = 0.645), Miru26 (HGDI = 0.616), and Qub26 (HGDI =0.608). Eleven loci (Mtub04, MIRU10, ETRE, MIRU39, ETRA, MIRU40, Qub4156c, Mtub39,Mtub30, ETRF, and Mtub38) showed moderate discriminatory power, and the remaining loci were less discriminative, with an HGDI ranging from 0.015 to 0.179. The discriminatory power (HGDI) of all the loci sets reached 0.998.

**Table 2:** Allelic diversity of 26 different MIRU-VNTR loci among *M.tuberculosis* strains (*n* = 668)

locus	HGDI (all strains)	HGDI (Beijing family)	HGDI (non-Beijing family)	Size range observed	24-loci VNTR	15-loci VNTR	12-loci VNTR	10-loci VNTR
qub-11b	0.767	0.728	0.845	0–11	√	√		√
mtub-21	0.644	0.564	0.762	1–8	√	√		√
miru-26	0.616	0.541	0.817	1–12	√	√	√	√
qub26	0.608	0.566	0.762	1–11	√	√		√
mtub-04	0.516	0.460	0.690	1–6	√	√		√
miru-10	0.451	0.357	0.639	1–6	√	√	√	√
ETRF	0.434	0.358	0.661	0–7				√
ETRE	0.430	0.312	0.410	1–5	√	√	√	√
miru-39	0.394	0.281	0.464	1–8	√	√		√
ETRA	0.329	0.236	0.565	1–5	√	√		√
miru-40	0.326	0.243	0.589	0–5	√	√	√	
qub4156	0.322	0.315	0.357	1–8	√	√		
mtub-38	0.322	0.110	0.602	0–3				
mtub-39	0.316	0.252	0.523	2–8	√		√	
mtub-30	0.308	0.168	0.502	2–5	√	√		
ETRB	0.179	0.097	0.468	1–5	√			
miru-27	0.175	0.117	0.413	1–4	√		√	
miru-16	0.172	0.120	0.396	1–5	√	√	√	
ETRD	0.159	0.094	0.436	0–6	√	√	√	
ETRC	0.109	0.097	0.172	2–5	√	√		
miru-23	0.109	0.090	0.203	1–6	√		√	
mtub29	0.096	0.077	0.187	2–6	√			
vntr49	0.081	0.080	0.088	1–4	√			
miru-20	0.030	0.028	0.036	1–2	√		√	
miru-2	0.015	0.014	0.018	1–3	√		√	
miru-24	0.015	0.007	0.054	1–3	√		√	
all	0.998	0.998	0.999					

We further compared the allelic diversity between the Beijing family and the total isolates. All 26 loci showed lower allelic diversity among the Beijing family isolates, which was in accordance with the closer affinity of this genotype (Table 2).

To evaluate the discriminatory power of different locus sets by MIRU-VNTR techniques, we compared the performance of this 26-locus set with the 24-locus, 15-locus and 12-locus sets coupled with spoligotyping (Table 3). These 26-locus, 24-locus and 15-locus sets obviously improved the performance compared with the initial 12-locus set, especially in combination with spoligotyping. The 12-locus set of MIRU-VNTR alone generated 263 genotypes, and the combination of 12-locus MIRU-VNTR and spoligotyping increased the number of genotypes to 292, resulting in HGDI values of 0.92 and 0.94, respectively. The 15-locus set generated 501 genotypes, and 15 clusters were subdivided in combination with spoligotyping, generating 516 patterns. The discriminatory power of the 15-locus set alone and combined with spoligotyping was 0.996 and 0.997, respectively. While the combination of the 24-locus set and spoligotyping yielded 561 genotypes with an HGDI value of 0.998, the 26-locus set with spoligotyping differentiated all the isolates into 576 different genotypes (HGDI = 0.998). In conclusion, both the 15-locus and 24-locus sets appeared to provide a discrimination power close to that of the 26-locus set.

**Table 3:** Number of different patterns and unique strains, clustering percentage, clustering rate and HGDI for the different typing methods in this study

Genotyping method	Number of pattern	Number of cluster	Number of clustered isolates	Number of isolates with unique profile	cluster rate (%)	HGDI
spoligotyping	35	10	643	25	0.948	0.325
12-loci miru-vntr	263	51	456	212	0.606	0.925
12loci + sp	292	53	429	239	0.563	0.941
15-loci miru-vntr	501	56	223	445	0.250	0.996
15loci + sp	516	54	206	462	0.228	0.997
24-loci miru-vntr	550	42	160	508	0.177	0.998
24loci + sp	561	40	147	521	0.160	0.998
26-loci miru-vntr	567	38	139	529	0.151	0.998
26loci + sp	576	37	129	539	0.138	0.999

To reduce the economic cost and lighten the labour load for high-throughput genotyping, we aimed to determine a minimal set of VNTR loci to analyse the isolates in Henan. We evaluated and compared the cumulative HGDI by successively adding each locus (Table 4). The 22-locus set produced the same cumulative HGDI and clustering rate as the 23-locus set (HGDI = 0.9983, clustering rate = 15.4%), and these two values of the 24- and 25-locus sets were also equal (HGDI = 0.9984, clustering rate = 15.2%). We identified the top 10 most discriminatory loci (qub-11b, mtub21, miru26, qub-26, mtub04, miru10, ETRF, ETRE, miru39 and ETRA) with a cumulative HGDI of 0.996. The combination of the top 10 loci showed adiscriminatory power close to that of the 26-locus set. There was no significant improvement in the cumulative HGDI upon adding other loci (Table 4).

**Table 4:** The cumulative HGDI with successive addition of each MIRU-VNTR locus

	VNTR locus	No. of pattern	No. of cluster	No. of cluster isolates	No. of isolates in each cluster	cluster rate (%)	HGDI (cumulative)
1	Qub11b						
2	mtub21	56	38	650	2–165	0.916	0.890
3	miru26	158	69	579	2–122	0.763	0.940
4	qub26	258	78	487	2–80	0.612	0.970
5	mtub-04	318	80	430	2–59	0.524	0.982
6	miru-10	356	84	396	2–49	0.467	0.988
7	ETRF	389	77	356	2–45	0.418	0.990
8	ETRE	420	72	320	2–42	0.371	0.992
9	miru-39	444	76	300	2–35	0.335	0.994
10	ETRA	466	72	274	2–28	0.302	0.996
11	miru-40	492	69	245	2–23	0.263	0.997
12	qub4156	507	63	224	2–21	0.241	0.997
13	mtub-38	512	62	218	2–21	0.234	0.997
14	mtub-39	524	55	199	2–21	0.216	0.997
15	mtub-30	528	53	193	2–20	0.210	0.997
16	ETRB	531	51	188	2–19	0.205	0.998
17	miru-27	536	50	182	2–18	0.198	0.998
18	miru-16	542	51	177	2–16	0.189	0.998
19	ETRD	548	46	166	2–15	0.180	0.998
20	ETRC	555	43	156	2–15	0.169	0.998
21	miru-23	562	42	146	2–15	0.156	0.998
22	mtub29	565	39	142	2–15	0.154	0.998
23	vntr49	565	39	142	2–15	0.154	0.998
24	miru-20	566	39	141	2–15	0.153	0.998
25	miru-2	566	39	141	2–15	0.153	0.998
26	miru-24	567	38	139	2–15	0.151	0.998

### Relationship between the Beijing family and drug resistance and sociodemographic characteristics

To assess the correlation between levels of drug resistance and genetic patterns, susceptibility to the four first-line and five second-line anti-TB drugs was determined with the proportional method. A total of 202 isolates (30.3%) were resistant to at least one drug, 50 (7.5%) isolates were classified as MDR-TB, and 30 isolates (4.5%) were susceptible to all four first-line drugs. As shown in Table 5, among the 50 MDR isolates, 47 showed the Beijing family genotype, and 3 showed the non-Beijing family genotype. In total, 8.4% (47/558) of the Beijing family genotype isolates were MDR, and 2.7% (3/110) of the non-Beijing family genotype isolates were MDR. For the Beijing family genotype, the percentage of isolates resistant to four first-line drugs was 5.19% (29/558), while only 0.91%(1/110) of the non-Beijing family isolates were resistant to four first-line drugs. Thus, the results revealed a significantly higher proportion of MDR among the Beijing family than among the non-Beijing family (OR 3.28, 95%CI 1.01–10.37, *p* = 0.038), and the Beijing family also showed a higher risk for developing drug resistance to all four first-line drugs (OR 5.97, 95%CI 1.05–44.33, *p* = 0.045).There was no apparent difference in the proportions of other drug-resistant profiles between the Beijing and non-Beijing family genotypes (Table 5).

**Table 5:** Differences in *M. tuberculosis* characteristics between the Beijing and non-Beijing families

Drug susceptibility testing	total (*n* = 668)	Beijing family isolates(*n* = 558)	Non-Beijing family isolates (*n* = 110)	OR	95% CI	*p*-value
Pan sensitive	466	394	72	1.09	0.69–1.75	0.700
Any resistance to						
Isoniazid	99	85	14	1.23	0.67–2.26	0.487
Rifampin	63	54	9	1.20	0.57–2.51	0.623
Ethambutol	42	38	4	1.56	0.54–4.50	0.415
Streptomycin	129	108	21	1.01	0.65–1.71	0.946
Kanamycin	28	20	8	2.11	0.90–4.92	0.078
ofloxacin	58	44	14	1.79	0.94–3.41	0.072
All four first-line drugs resistance	30	29	1	5.97	1.05–44.33	0.045
MDR-TB	50	47	3	3.28	1.01–10.37	0.038
Pre-XDR-TB	17	16	1	3.21	0.42–24.51	0.332
XDR-TB	11	10	1	1.99	0.25–15.69	1.001

*OR* odds ratio, *CI* confidence interval

MDR-TB: multi-drug resistance TB, resistant to at least isoniazid and rifampicin

XDR-TB: extensively drug-resistant tuberculosis, MDR-TB plus resistance to ofloxacin and kanamycin

Pre-XDR-TB: MDR-TB plus resistance to either ofloxacin or kanamycin

First-line drugs resistance: resistant to isoniazid, rifampin, ethambutol and streptomycin

Then, the sociodemographic characteristics of patients, including gender, age at diagnosis, and clinical history, were compared between the Beijing and non-Beijing family genotypes, and no statistically significant differences were detected (Table 6).

**Table 6:** Epidemiological characteristics of the Beijing and non-Beijing isolates

characteristics	total (*n* = 668)	Beijing family isolates (*n* = 558)	Non-Beijing family isolates (*n* = 110)	OR	95% CI	*p*-value
Sex						
Male	509	428	81			
Female	159	130	29	0.85	0.53–1.35	0.541
Age group,years						
< 25	141	113	28			
25–44	197	161	36	0.71	0.39–1.31	0.282
45–64	177	154	23	0.79	0.45–1.40	0.422
> 64	153	130	23	1.19	0.63–2.20	0.587
Treatment history						
New case	501	422	79			
Retreatment case	167	136	31	1.22	0.77–1.92	0.398
Population						
Permanent	134	111	23			
Migrant	534	447	87	1.06	0.64–1.76	0.804

## Discussion

The Beijing family genotype remains the predominant genotype in China [14]; however, the proportion of patients carrying this genotype varies in different regions [14]. Henan Province has a high incidence of tuberculosis, but little is known about the genetic background of *M. tuberculosis* in this region. This is the first study to investigate the allelic diversity of MTB isolates in Henan Province using spoligotyping and MIRU-VNTR.

*M. tuberculosis* is divided into 162 clades according to the international spoligotyping database SpolDB4 [26], and the Beijing family is regarded as the most important genetic pattern of the East Asian clade [22]. The pattern is predominant in China, and its proportion is greater in northern China than in southern China. The prevalence rate of the Beijing family is higher in Beijing (82–92.6%) [31, 33], Tianjin (91.7%) [34], Tibet (96.3%) [33, 35], Inner Mongolia (93.3%) [36], Heilongjiang (89.5%) [37],and Gansu(87.5%) [38] but lower in Guangdong(25%) [39], Guangxi (55.3%) [35] and Fujian(54.5–55.1%) [33, 40]. These results showed that 83.5% of the MTB isolates belonged to the Beijing family, indicating that this genotype was the most predominant genotype in our region, which was consistent with the results of previous studies. The higher prevalence of the Beijing family might be associated with customs influenced by climate [14]. The non-Beijing family lineage included the T1, T2, T3, MANU1, MANU2, S, LAM3,S/convergent, LAM3/S and U genotypes, indicating genotypic polymorphism among MTB strains in this area. Among the non-Beijing family isolates, 59 were in the T1 family (53.6%), 20 were in the MANU2 family (18.2%), 10 were in the T3 family (9.1%), and 7 were in the T2 family (6.4%); these genotypes have also been observed in other regions of China, albeit at different proportions [35–40]. This study also identified eight new spoligotype isolates. They were divided into different clusters and were derived from patients in different regions, indicating there might not show an epidemiologic relationship. The larger number of small gene clusters could potentially reflect a recent transmission. The higher rate of small genotypic clusters for the Beijing family suggested its predominance in recent transmissions.

The prevalence of the Beijing genotype is apparently higher worldwide, but very little is known about the reasons for its efficient transmission. Previous studies have suggested that this genotype is associated with drug resistance and shows increased virulence in animal models [41] and enhanced reproductive fitness [42]. Overproduction of polyketide synthase-derived phenolic glycolipid (PGL) by the Beijing family inhibits the release of pro-inflammatory cytokines, thus enhancing the infective success [43, 44]. The Beijing family has a strong association with drug resistance, indicating that this family might be predisposed to acquiring resistance [45] and thereby showing increased transmission of drug-resistant *M.tuberculosis*. However, there has been a discrepancy in different research results for the non-Beijing family because this family includes various subtypes. There is a discrepancy in the relationship between the Beijing lineage and TB outbreaks in a variety of geographic locations [23, 45–52].To analyse the relationship between the Beijing family and drug resistance, we compared the proportion of Beijing genotypes in different drug susceptibility profiles, and the results showed that resistance to all four first-line drugs was significantly higher in the Beijing family. The Beijing family isolates had a higher resistance rate to INH, RIF and MDR in Ukraine [53]. Similarly, the Beijing family also had a close association with INH, RIF, SM and MDR resistance in Central Asia [54]. In agreement with previously reported data, our data revealed that the Beijing genotype showed a greater correlation with MDR-TB phenotypes than did other non-Beijing genotypes. The long-term reciprocal co-evolution between host and bacterium might affect the prevalence of the Beijing genotype [55, 56], thus, we estimated the correlation between the Beijing family and epidemiological features, including sex, age and treatment status. Previously, some studies revealed that the Beijing genotype strains are generally associated with young age [57] and a higher rates of treatment failure and relapse than other strains [58, 59], but in this study, there was no association between the prevalence of Beijing genotypes and gender, age or clinical treatment history of patients. Therefore, it is necessary to further explore the effect of demographic factors on the genetic diversity of *M. Tuberculosis* with a larger sample size in our area.

Spoligotyping is an efficient genotyping technique that can classify the MTB lineage, but it cannot effectively distinguish Beijing family isolates due to lower discriminatory power [35]. In this study, 668 samples were successfully classified into 35 distinct genotypes, including 10 clusters and 25 unique spoligotypes. Due to the low resolution of spoligotyping, we applied another typing method based on MIRU-VNTR to further phylogenetically analyse the molecular characteristics of these isolates [35, 37]. It is important to choose the appropriate VNTR loci to identify the most prevalent cluster for the Beijing family. The classical 12-locus MIRU-VNTR set is a widely used molecular epidemiological approach to elucidate the phylogenetic diversity of MTB isolates, but it is not effective at distinguishing Beijing isolates. The 15-locus and 24-locus VNTR combinations have sufficient discriminatory power and are suitable for MTB genotyping, especially in areas where the Beijing family is prevalent. However, it is not necessary to utilize all 24 loci for genotyping MTB isolates due to the diversity of the isolate population structure. In addition, the 24-locus set is very time consuming and complicated to operate. The genotyping efficiency varied depending on the disparate loci sets in different surveyed areas. To identify a suitable locus set for classifying MTB in Henan, we first chose the 26-locus set to assess the 668 clinical isolates according to previous studies [21, 22, 60].In total, 567 genotypes, forming 38 clusters, and 529 unique genotypes were obtained by 26-locus VNTR analysis with an HGDI score of 0.9984. For the Beijing family isolates, the clustering rate (16.12%) of the 26-locus set was obviously lower than that of spoligotyping (98.74%), and the cumulative HGDI value (0.998) of the 26-locus set was significantly higher than that of spoligotyping (0.042). Moreover, the clustering rate of VNTR was different between Beijing and non-Beijing families (16.13% vs. 2.7%), suggesting that the Beijing family may have more effective infectivity in Henan. Previous studies showed that the combination of MIRU-VNTR and spoligotyping could enhance the discriminatory power of MTB [19, 61].Correspondingly, our data showed that the combination of spoligotyping and the 26-locus set VNTR finally classified all 668 isolates into 576 different patterns and had a lower clustering rate (13.77%) than that with 26-locus VNTR (Table 4).

Our analysis showed that 11 loci of the 26-locus MIRU-VNTR set were poorly discriminatory. Especially, the VNTR49 and MIRU02 loci did not improve the cumulative HGDI, indicating that these two loci were conserved, resulting in no power to discriminate different MTB isolates. In this study, the two largest clusters contained 15 and 14 strains, and the other clusters were composed of 2 to 9 strains. Among 29 isolates of the two largest clusters, 24 (82.7%) belonged to the Beijing family, three to the T1 family, one to MANU2, and one to the H3 family. Moreover, 10 (34.5%) of these 29 isolates were resistant to one or more drugs. However, it remains uncertain whether the patients infected with these strains had close contact with each other. Since the ability of the different locus sets to classify MTB was diverse in Henan, we needed to determine an optimal set that had a discriminatory power comparable to that of the 26-locus set. In consideration of labour and economic costs, we chose the top 10 loci combination, which had an HGDI value comparable to that of the classical 24-locus set (0.996 vs. 0.997), which was slightly lower than that of the 26-locus set (0.998). These data indicated that the 10-locus combination was useful and cost-efficient. Therefore, we suggest this 10-locus set as a potential first-line MTB genotyping method in Henan Province, especially for a large-scale molecular epidemiological survey.

Our data revealed some inconsistency between the results of spoligotyping and MIRU-VNTR for several MTB isolates. Beijing family isolates comprised a large proportion of the largest cluster by MIRU-VNTR genotyping; however,33 isolates with a non-Beijing spoligotype were found in this largest clade. Furthermore, another 19 strains showed the Beijing genotype spoligotype pattern but could not be distinguished by MIRU-VNTR (Fig. 1).This divergence might be due to mixed infection of two different TB isolates in these samples [62].

Until now, no genotyping methods based on genetic markers have been able to completely accurately classify the Beijing family because there were always exceptional strains [46]. Previous studies showed that different VNTR loci had varying discriminatory power for the Beijing and non-Beijing family genotypes [21, 63]. In our study, only Qub11b had a higher discriminatory power for the Beijing family among 26 loci. However, six loci (loci qub-11b, mtub-21, miru-26, qub26, mtub-04 and miru-10) exhibited a higher discriminatory power for the non-Beijing family. Four loci (Mtub38, ETR-B, ETR-D and MIRU40) showed remarkable differences in allelic diversity between Beijing and non-Beijing genotypes, with the difference in the HGDI greater than 0.25.

There are several limitations of this study. First, the small sample size is a major limitation. Further in order to ensure greater reliability and representativeness of the findings, we should enlarge the sample for further observation in the future. Furthermore, the initial isolates taken by sputum were not collected when retreated tuberculosis patients were first diagnosed in this study; thus, we were unable to differentiate relapse and reinfection cases.

## Conclusion

We report the genotype distribution of *M. Tuberculosis* strains in Henan Province. Based on our results, the combination of spoligotyping and 26-locus VNTR can effectively analyse the molecular epidemiological features of MTB in this area. The Beijing genotype was the predominant genotype in this area and exhibited a great correlation with multi-drug-resistant phenotypes. The analysis of MIRU-VNTR data can help select the appropriate VNTR loci to genotype MTB in specific regions; our results identified a reduced 10-locus MIRU-VNTR set that could be applied to distinguish most MTB lineages in this region. In future studies, superior discriminatory power methods, such as genome sequencing, will be used to classify the remaining clusters based on MIRU-VNTR to better understand their significance related to ongoing TB transmission. Overall, this study provided an effective method to distinguish MTB isolates, and the implementation of this technology will help enhance TB control programmes and reduce the TB burden in Henan Province.

## Additional file

Additional file 1: **Figure S1.** Genotyping of 668 *M. tuberculosis* isolates with 26-locus MIRU-VNTR and spoligotyping. The clustering was based on the analysis performed using BioNumerics 6.6 to compare these two genotyping methods. From left to right: (1) UPGMA dendrogram generated by the 26-locus MIRU-VNTR, (2) spoligotyping patterns, and (3) strain number. (PDF 113 kb)

## Abbreviations

CPM: Capreomycin
DRs: Direct repeats
EMB: Ethambutol
HGDI: Hunter and Gaston Discriminatory Index
INH: Isoniazid
IS6110: Insertion sequence 6110
KM: Kanamycin
L-J media: Lowenstein-Jensen media
LSP: Large sequence polymorphism
MDR-TB: Multidrug resistance- TB
MIRU-VNTR: Mycobacterial interspersed repetitive unite variable number tandem repeat
MTB: *Mycobacterium tuberculosis*
OFX: Ofloxacin
PAS: p-aminosalicylic acid
Pto: protionamide
RFLP: Restriction fragment length polymorphism
RIF: Rifampicin
SIT: Spoligotype International Type
SM: Streptomycin
spoligotyping: Spacer oligonucleotide typing
TB: Tuberculosis
WHO: World Health Organization
XDR-TB: Extensively drug-resistant tuberculosis
