# Supplementary figures and images for: Role of MIRU-VNTR and spoligotyping in assessing the genetic diversity of Mycobacterium tuberculosis in Henan Province, China
Source: BMC Infect Dis. 2018 Sep 3;18:447. doi: 10.1186/s12879-018-3351-y (PMC6122615; doi:10.1186/s12879-018-3351-y)

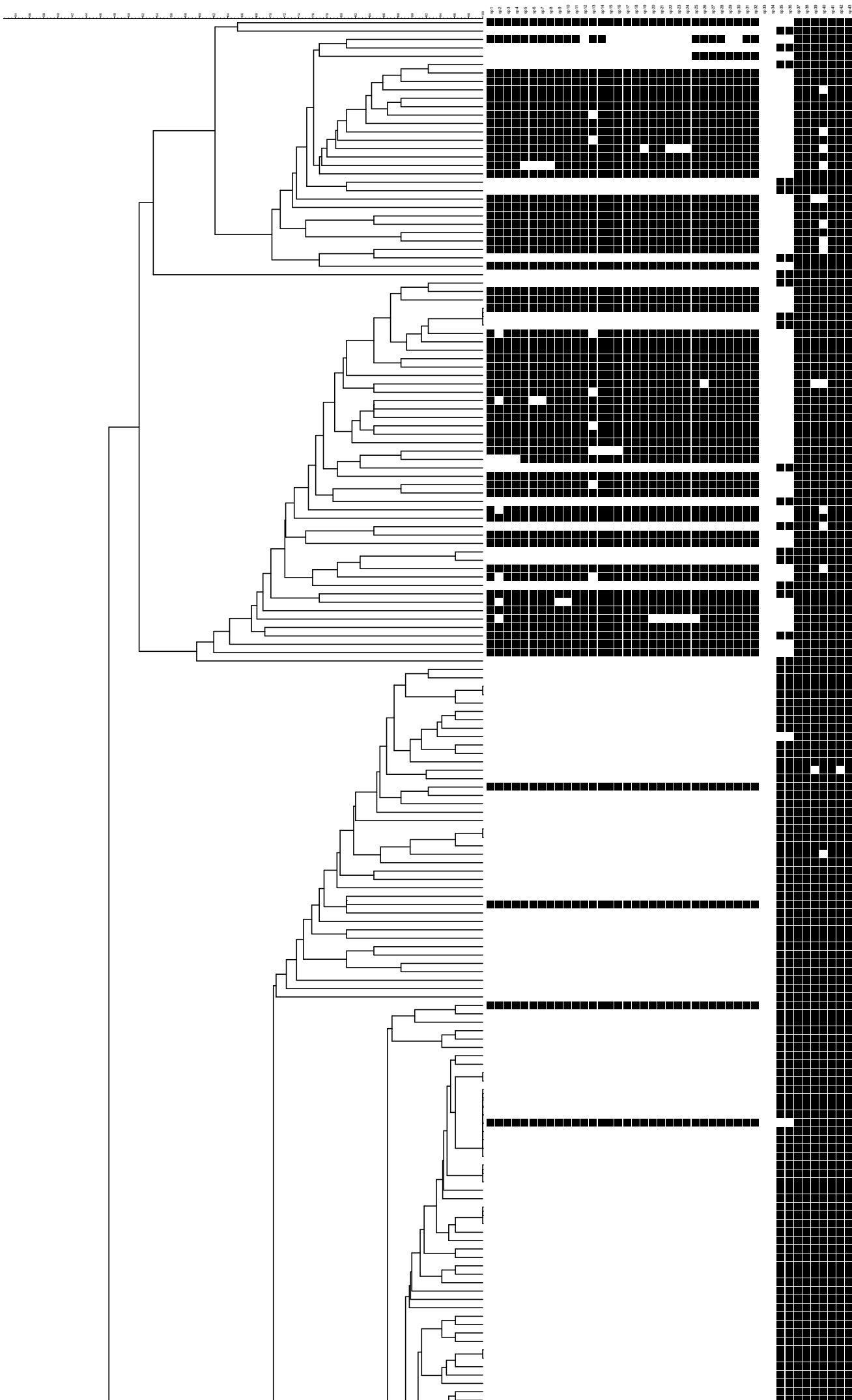

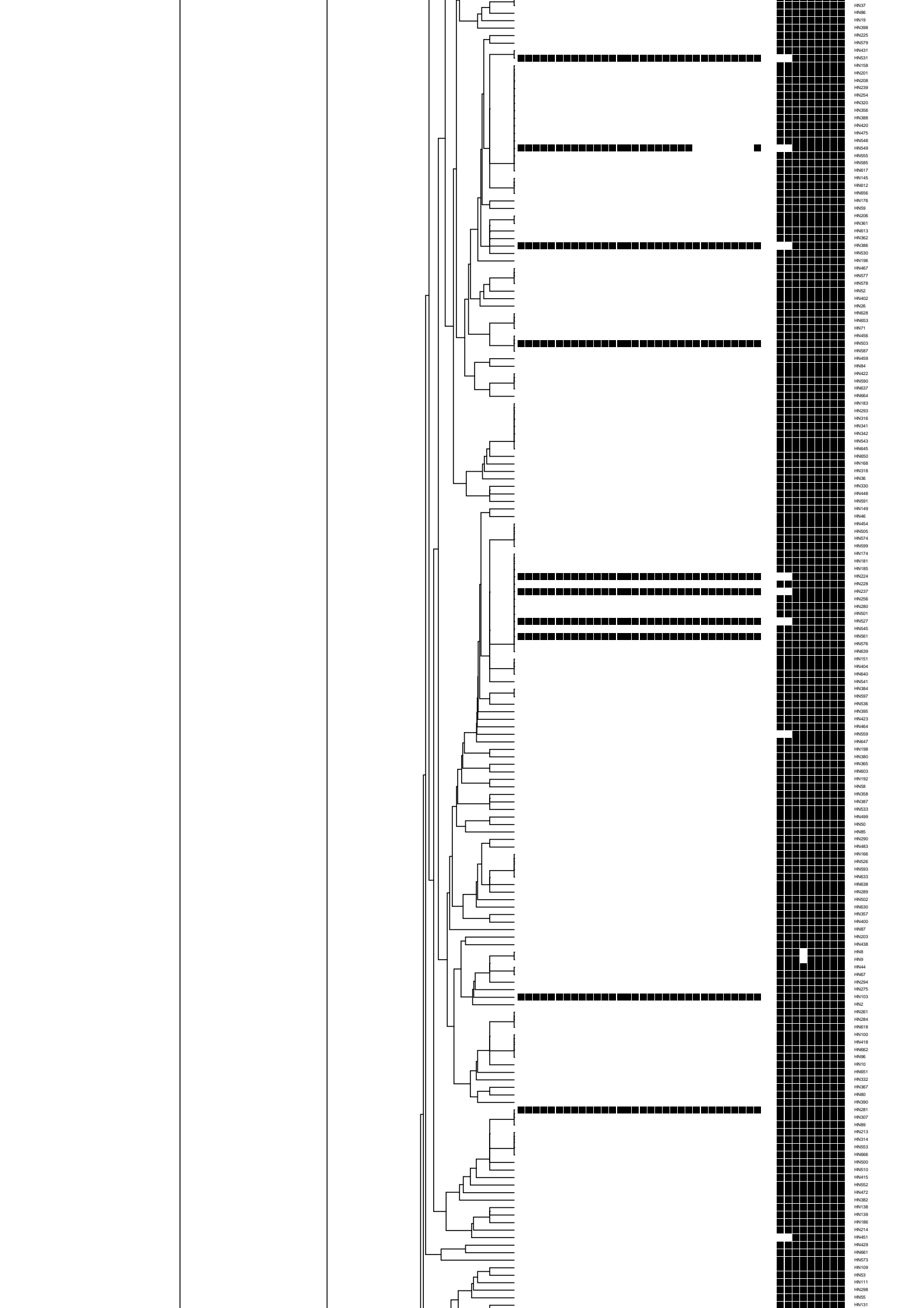

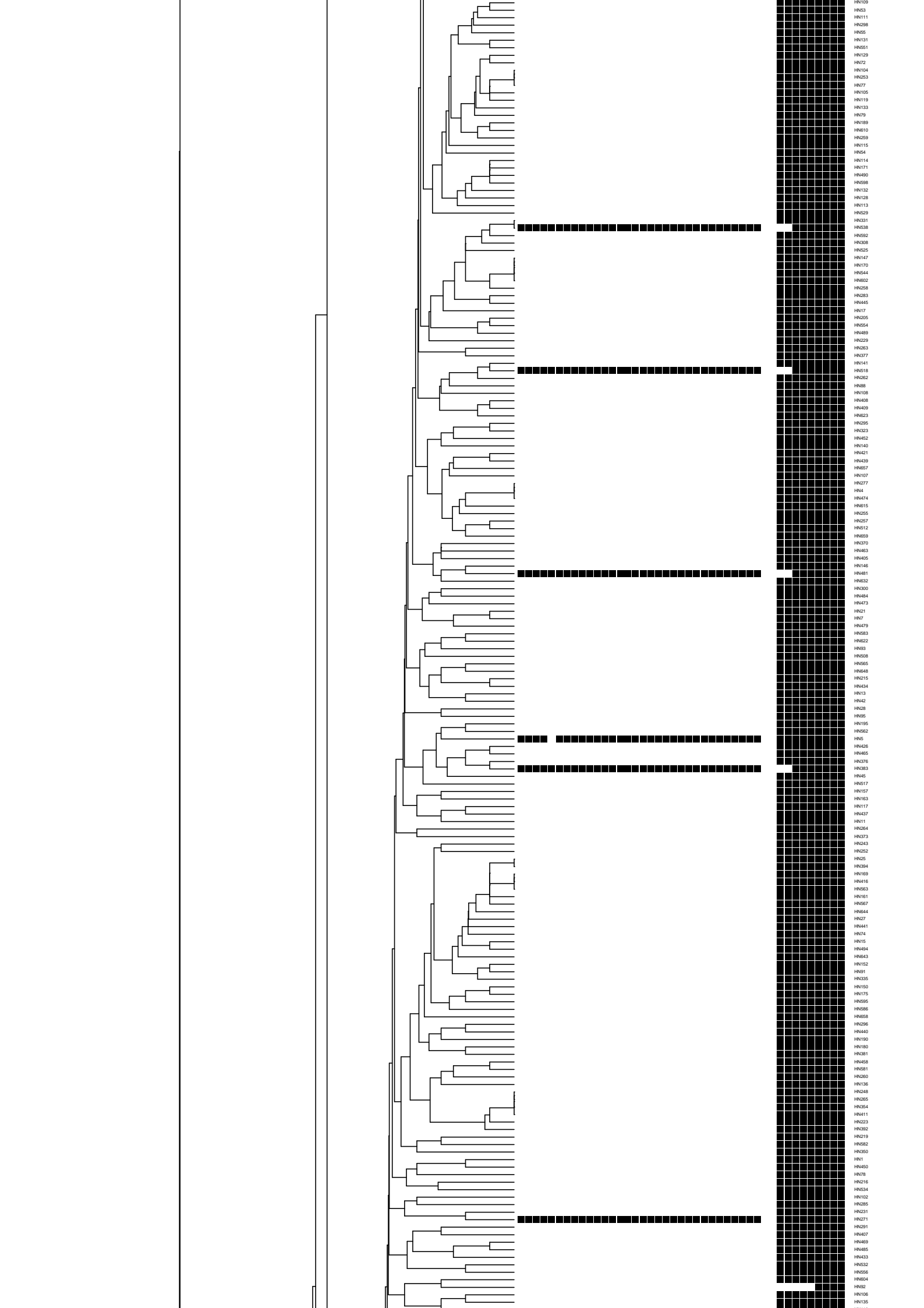

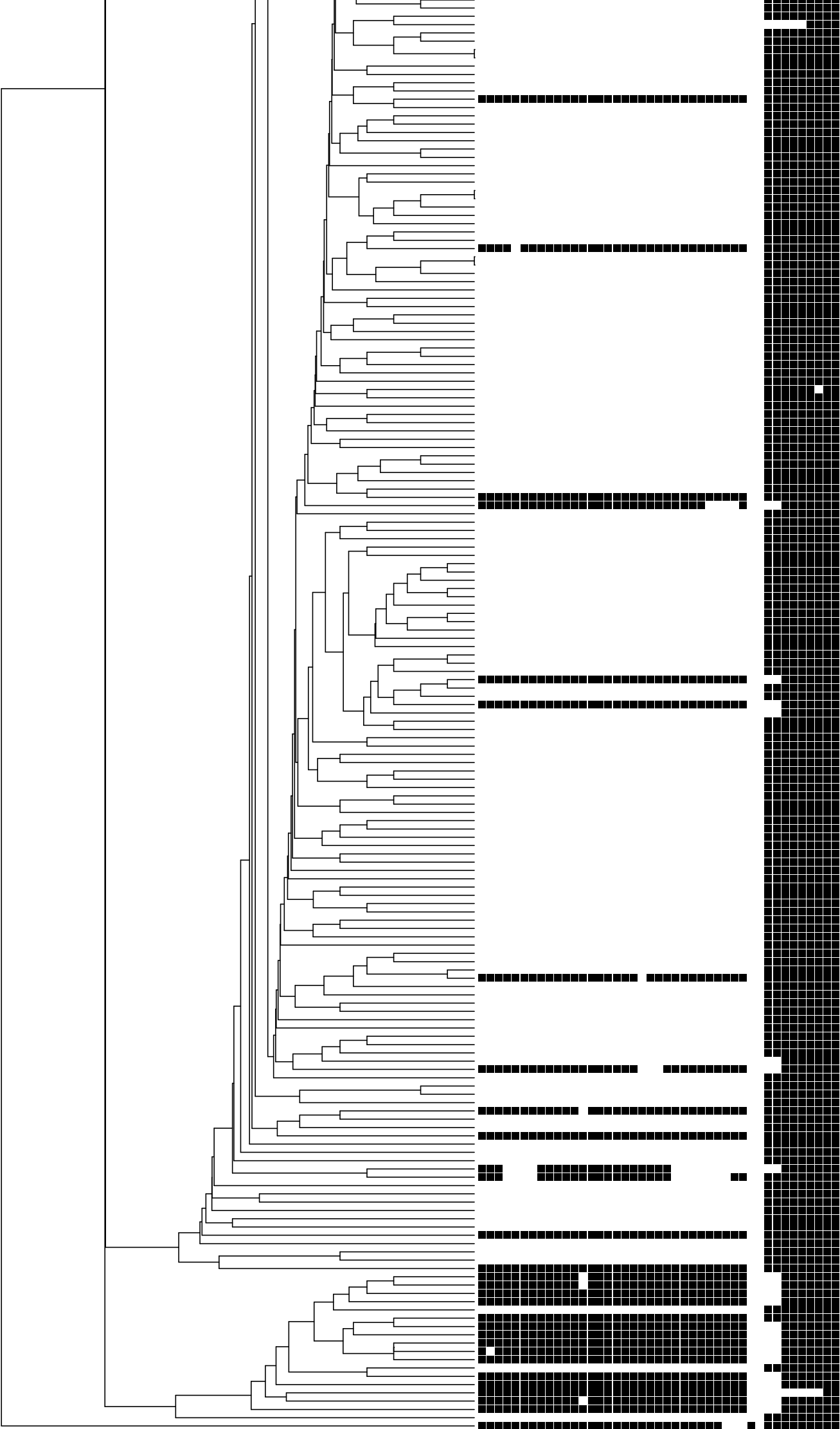

Supplement: Supplementary file 1 — Figure S1. Genotyping of 668 M. tuberculosis isolates with 26-locus MIRU-VNTR and spoligotyping. The clustering was based on the analysis performed using BioNumerics 6.6 to compare these two genotyping methods. From left to right: (1) UPGMA dendrogram generated by the 26-locus MIRU-VNTR, (2) spoligotyping patterns, and (3) strain number. (PDF 113 kb) [file 12879_2018_3351_MOESM1_ESM.pdf]
